# A Case of Apoplexy

**Published:** 1868-10-01

**Authors:** Curtis T. Fenn

**Affiliations:** 234 Thirty-first Street; Chicago


### A CASE OF APOPLEXY.

BY CURTIS T. FENN, M.D., CHICAGO.

THE following is the history of a case of apoplexy which I witnessed from the moment of attack. The subject, aged sixty-one years, was a native and resident of Vermont, once a man of apparently good constitution, thick set, rather fleshy, a farmer, married, without children, of laborious habits, a hearty eater, given to the habitual use, though not abuse, of hard cider. Apprehension that his health was failing, induced him to resign all care two years ago. The pursuits of a large farm had never satisfied him; but at this time he grew quite restless. He acquired a belief that his heart was diseased, and that he might die suddenly. This increased his unhappiness. Feeling the need of more cheerful surroundings, he went abroad, but returned in a few months more than ever depressed. He experienced fatigue on slight exertion; his pulse was uniformly too rapid; and his heart's action often tumultuous; his breathing was short; his lungs inclined to fill up. He had no pain; he thought his mind as comprehensive and clear as ever; his vision and hearing were good; he invariably slept well — too well he thought; his appetite remained excellent; his bowels regular. He was annoyed by thirst, desiring to drink immoderately of water, but from this he refrained; his urine was of proper quantity and color; he perspired easily; his extremities were apt to become cold; his face was sometimes flushed, and at other times pale; occasionally he took opium in small quantity, which made him "feel *stronger* and *happier — it relieved his heart.*"

I learned these facts from him about noon, June 9, 1868. At the same time the area of the heart's dullness was ascertained to be greatest in its transverse direction; the apex beat feebly, and felt over two or three intercostal spaces, extending to the left of its normal place; the pulse was rapid, soft, and full, 108 per minute; half an hour later it had fallen to 101; the sounds of the heart were indistinct and short, seeming to unite in the first sound, which had the character of the second; respiration was occasionally sighing; his chest was full and resonant; râles were present throughout, and coughing was accompanied by expectoration of clear, frothy mucus; his tongue was coated with a thick, yellowish, pasty fur; gums spongy, and encroached upon by tartar, in spots; abdomen large, having, numerous dilated vernicles upon the surface; skin soft and moist; eye natural; top of head bald.

He was a little excited during the examination, and said that it affected him, but at the close he appeared cheerful, and proposed exercise — said he had walked four miles the day before, and felt the better for it. He asked what I thought of the probability of apoplexy in his case, dwelling upon instances of death from that disease, which he had known. His mother died of it, with general paralysis, at seventy; his youngest brother had something like it at forty. I endeavored to turn his thoughts upon pleasanter themes. The day was hot and sultry. We took our course along a new portion of the city, and having gone, perhaps, three miles, with occasional intervals of rest, were returning home. I thought he seemed in a hurry, disposed to silence, less observant. After a time he asked if we were almost there. In answer to the inquiry if he felt tired, he said his *left* leg was numb. I suggested that he sit down. He said no, and continued his step. His breathing was excited, and his face pale. A little further on he paused and held up his right foot, flexing and extending it, and then stepping, as if to be assured of its integrity; he said it was a strange sensation which he felt; he had never experienced any thing like that before. I urged him to stop. He resumed his walk, making little exclamations of surprise. I supported his left arm. He seemed determined on walking faster, but soon began stubbing his right foot at every step, inclined to fall forward. His right knee presently bent beneath the weight of his body, and gradually refused to bear him. Still he hobbled, dragging the leg. I could no longer prevent his falling; he reeled toward the right, and sank down upon the plank sidewalk. I placed him sitting while he endeavored to remove his right boot, using his left foot and hand — his right arm hung loosely by his side. I suggested rubbing his leg, telling him not to be frightened. He said nothing, but looked vacantly along the street. I asked him why he complained of his left leg's being numb, when it was his right. He did not know that he said left His pulse was regular, full, soft, not so rapid as when observed before. Perspiration fell from his face. When asked if he felt better, he said he guessed so.

A gentleman passing kindly assisted to get him into his carriage, and in a few minutes we lifted him to the residence whence he had walked so confidently a couple of hours before. His face was sunken and haggard, but in no way distorted; his right side was powerless, and deprived of sensation. His mind did not appear bright, though he articulated correctly. When laid upon a lounge he yawned repeatedly, and asked with non-concern if it was a good time for him to sleep, saying that he felt sleepy. The pupils were unaffected. He uttered one other articulate expression and immediately went to sleep. His pulse was regular, full, soft, 100 per minute; respiration irregular; extremities cool. He was easily roused, answering to the point, but sank into more and more profound slumber. He evacuated urine incontinently, while asking for assistance. He was put to bed in a cool room, his head slightly raised and ice applied. He had eaten less than usual that morning, and tasted no food since. A saline laxative was given, and hot bottles applied to his feet.

Dr. Bevan came to my assistance five hours after the attack. Coma was then complete. His pulse had fallen to 90, regular, soft, full. Respiration irregular, through the open mouth, stertorous; pupil contracted. It was ordered that a grain of *Calomel* be given every four hours till his bowels should move freely, and sinapisms applied to his feet and legs. Not much change appeared through the night; the pulse remained at about 92, soft and full; respiration irregular, 11 to 16 per minute. After a full breath there followed respirations slower and less complete successively, till they ceased altogether for an interval of fifteen seconds, to be renewed by a full long drawn breath, accompanied by stertor.

*Second day.* Face flushed, surface of body hot; pulse 109, rather harder; respiration as before, pupil contracted; breath fœtid. P. M. Increasing consciousness. Night pulse 116, steady, improved consciousness; whenever roused he continued to yawn, remained awake a few minutes, then fell again into deep sleep; took three cups of oatmeal gruel through the day, swallowing without difficulty; no paralysis of any part of face visible; a spasmodic movement of the right knee observed while he was asleep; urine voided involuntarily; tested, it gave strong acid reaction and considerable quantity of albumen.

*Third day.* Pulse 96; respiration regular; continued through open mouth; consciousness further improved; when awake answered questions correctly, with some obstruction; left angle of mouth slightly elevated; tongue protruded straight; pupils nearly normal; right eye watery, the lid not closing perfectly; inclined to lie upon his back or right side. A half ounce of *Sulphate of Magnesia* induced a copious evacuation of the bowels. The *Bromide of Potassium* was commenced in 20 grain doses, to be given every four hours, and beef tea through the day. Noon: Pulse 118. He grew more and more feverish and restless till night; occasionally muttering delirium. The *Tincture of Digitalis* was given in fifteen drop doses. 9 P.M.: The skin was hot and dry. A Dover's powder of ten grains was succeeded by quietude during the night.

*Fourth day.* Pulse 90; respiration regular; face pale; he tried to get up, evincing no surprise that he could not; asked where we were; when moved in bed complained; right thumb and fingers slightly drawn into the palm. P.M.: Pulse 124, harder; increased heat, perspiration and lividity; respiration accompanied by tracheal râles which did not excite coughing. *Digitalis* and *ice* continued without diminution of excitement. Night passed restlessly; bathed in perspiration.

*Fifth day.* Pulse 108, weak; respiration 24; expectoration streaked with blood, thick, fœtid. He appears quite animated, asked for friends and tried to leave his bed; right cheek flaccid and opposite angle of mouth more drawn up; had an incontinent movement of the bowels; there appeared a bright red spot, two or three lines in diameter, within the right conjunctiva; tongue dry and dark. P.M.: Pulse 144; restlessness and perspiration not less than day before. Wine was given every four hours. Night: Pulse intermittent, losing one or two beats a minute. A sixth of a grain of *Sulphate of morphia* was given at bed time; sound sleep followed.

*Sixth day.* Weaker than ever; rational; pulse 104, regular; perspiring freely. Noon: Pulse 118; 1 P.M., pulse 126; 2.30 P.M., pulse 132; 6 P.M., pulse 140; 7 P.M., pulse 144; deglutition difficult through the day. Little food or medicine administered.

*Seventh day.* Pulse 122; very drowsy; prostration marked; perspiration continuing. P.M.: Breathing labored; face constantly livid. Night: Pulse 152; respiration 50; comatose; pupil again contracted to size of a pin's head; tongue dry and brown; slight tympanitis; skin bathed in perspiration, relaxed, pale.

*Eighth day.* 3 A.M.: Profound coma; pulse 153; respiration 54; laryngeal râles. At 9 A.M. he died.

It is to be observed that there were no premonitory symptoms. The onset was sudden, the progress gradual. There was at first numbness, weakness, and slight confusion of ideas; then paralysis, anæsthesia and stupor; lastly insensibility. The first interval was perhaps five minutes, during which he continued on his feet; twenty minutes later he began to sleep; in five hours he was deeply comatose.

Amelioration of symptoms was present every morning, and, after the second day, increased excitement every evening—frequent pulse, passive congestion of head and face, heat of skin, perspiration, restlessness and muttering. Next morning ensued tranquility and improved consciousness, but with them came extended paralysis. These exacerbations were more marked each day, the reactions being less and less satisfactory; on the seventh day there was a return of the worst apoplectic symptoms, ending in exhaustion and death.

Hæmorrhage to considerable extent within the left hemisphere was no doubt the exciting cause. Mental agitation, heat and exercise, may have determined it; but back of all existed disease. An autopsy was impracticable. We must rely upon the antecedent history to reveal the primary affection.

There had been marked debility for two years at least, induced amid external conditions the most favorable to healthy nutrition. Materials were prepared in abundance. There must have been, therefore, a defect presiding over the formative cell changes; and, necessarily then, degeneration. The tissues became relaxed, softened, fatty; hence the muscular debility, disease of the kidneys, enlargement of the heart, and fragility of the capillaries; hence the apoplexy.

This defect of nerve force arose from a central lesion perpetuating itself. He had always allowed himself to be neglectful of important matters of business, and careless of every thing around him, a trait dependent on inability to keep his mind on one subject very long at a time. Respected by all for his good deeds and sound judgment, he was disposed to melancholy and difficult to entertain. After a life of extreme moderation in the employment of the generative function, its power wholly ceased four years ago. From that time all his mental peculiarities grew more striking. These facts, gathered after the death, confirm us in the belief that organic disease of the brain had existed long.

Finally, this local manifestion of some morbid condition may have been congenital. One cousin by the mother's side died insane; another is an imbecile. Considering also the circum stances of the mother's death, we are urged to believe that the original departure affected the constitution from birth, was indeed impressed upon the germ at conception.

Thus we have endeavored to sketch the natural history and progress of one form of apoplexy. Of course no treatment which does not look to the support of the patient, either before or after the accident of the hæmorrhage, can be of any avail.

234 THIRTY-FIRST STREET, *September* 10*th*, 1868.

